# Movie editing influences spectators’ time perception

**DOI:** 10.1038/s41598-022-23992-2

**Published:** 2022-11-22

**Authors:** Klara Kovarski, Joanna Dos Reis, Claire Chevais, Anaïs Hamel, Dominique Makowski, Marco Sperduti

**Affiliations:** 1 Hôpital Fondation Rothschild, Paris, France; 2 Integrative Neuroscience and Cognition Center-CNRS, Université Paris Cité, Paris, France; 3 Sorbonne Université, INSPE, Paris, France; 4 LaPsyDÉ, Université Paris Cité, CNRS, Paris, France; 5 Laboratoire Mémoire, Cerveau and Cognition, (LMC2 UPR 7536), Institut de Psychologie, Université Paris Cité, 92100 Boulogne-Billancourt, France; 6 Normandie Univ, UNICAEN, INSERM, U1237, PhIND “Physiopathology and Imaging of Neurological Disorders”, Neuropresage Team, Institut Blood and Brain @ Caen-Normandie, Cyceron, Caen, France; 7 UR2NF-Neuropsychology and Functional Neuroimaging Research Unit at CRCN-Center for Research in Cognition and Neurosciences and UNI - ULB Neuroscience Institute, Université Libre de Bruxelles (ULB), Brussels, Belgium; 8 Clinical Brain Lab, Nanyang Technological University, Singapore, Singapore

**Keywords:** Human behaviour, Attention

## Abstract

Filmmakers use different techniques (e.g., camera movements, editing) to shape viewers' experience. In particular, editing can be used to handle the temporal unfolding of events represented in a movie. Nevertheless, little is known about how different editing types impact viewers’ time perception. In an exploratory on-line study (90 participants) and a pre-registered conceptual replication study (60 participants), we asked participants to judge (Study 1) or reproduce (Study 2) the duration of 45 excerpts of the movie “*Le Ballon Rouge*” containing either continuous editing, action discontinuity editing or no editing. Each excerpt was formatted in three durations (2000, 2500 or 3000 ms). In both studies, we reported that scenes containing continuous editing were perceived as longer than the other two scene types. Moreover, scenes containing action discontinuity editing were perceived as longer than scenes with no editing. This study contributes to the emerging field of psycho-cinematics which could ultimately develop the dialog between arts and science.

## Introduction

The dialogue between arts and psychology has gained attention, by enhancing the understanding of how artistic objects are processed and perceived and how artists influence and create specific perceptual phenomena^1^. Concurrently, using artistic objects as stimuli also allows better apprehending of human behavior and brain functioning. Among others, this dual interest in this new interdisciplinary field has been flourishing in developing the relation between cinema and cognitive science. Films can be used by researchers as complex stimuli to study several cognitive processes such as natural vision^2^ or memory formation for complex material^3^. In particular, studying spectators’ cinematic experience could lead to crucial understanding on perceptual processes, since filmmakers have often developed a theory of perception through their intuition and a true experimental approach, a paradigmatic example of which is the Koulechov effect. This editing effect consists in attributing an emotional or a motivational content to a neutral face followed by another photograph. This effect is surprisingly close in psychology to priming, and has recently been exploited and reproduced in neuroscientific studies, suggesting that editing can be used to investigate several perceptual processes^4^.

More than other forms of art, cinema deals with time and duration perception^5^. Among other cinema language rules, editing rules have been established to assure syntactic and semantic continuity, but also to deal with rhythm (i.e. pacing). The most current editing type is the cut^6^, consisting in the juxtaposition of shots (i.e. montage), leading to perceptual discontinuity and the possible detection of an event. Continuity editing allows maintaining a continuous and narrative action^6^. To provide this effect, shots are matched across boundaries, along perceptual features, spatiotemporal relations, and actions.

Interestingly, empirical studies have shown that cuts following continuity editing rules pass unnoticed between a quarter and a third of the time, a phenomenon named “edit blindness”^7^. This effect seems to be driven by the modulation of the attentional dynamic induced^8,9^ by cuts toward the narrative, and away from low-level perceptual information^10^. In addition to editing rules, different styles, varying in the density of cuts and length of shots, are observed in cinema language. We will use the terms of editing or cut types to refer to the rules used for juxtaposing shots (e.g. continuous editing), and editing styles for rules concerning the organization of the cuts (or their absence) in the movie (e.g. action movies, continuous take). Editing techniques are specifically employed to handle the discrepancy between the temporal unfolding inside the narrative (diegetic time) and the real duration of the narrative^11^.

Surprisingly, to our knowledge, there is only one published study investigating how editing style modulates spectators’ time perception. Balzarotti, et al.^12^, asked participants to judge the duration of different video clips representing goal-directed routine actions. The authors manipulated the editing density (number of cuts) of video clips to obtain three different versions: a master shot (without cuts), a slow-paced editing (5 cuts), and a fast-paced (10–12 cuts). They showed that the duration of fast-paced video was overestimated compared to the master shot. It is important to note that both slow- and fast-paced video were created respecting continuity editing rules. Nevertheless, different kinds of cuts could differently affect cognitive processes and consequently time perception.

Magliano and Zacks^13^ investigated how different degrees of continuity/discontinuity at editing points modulate how individuals perceive and segment events while watching a film (Le Ballon Rouge by Lamorisse, 1956). In particular, they identified three types of editing: edits that are continuous in space, time, and action; edits that are discontinuous in space or time but continuous in action; and edits that are discontinuous in space and time as well as in action. Cuts associated with action discontinuity more robustly predicted the probability of detecting an event boundary. Event Segmentation Theory^14,15^ predicts that an event boundary is detected when perceptual predictions based on the actual active event model (a working memory representation of what is occurring in a given situation) are no longer accurate. At this point, a gating mechanism transiently increases the bottom-up influence of sensory information over the event model to up-date it. Thus, event segmentation implements an attentional mechanism that is meant to efficiently allocate cognitive resources over time to bias perceptual processing of pertinent information^15^.

Crucially, attention is a central feature of classical cognitive models of time perception^16–18^. Briefly, these models put forward the existence of an internal clock composed by a pacemaker, an accumulator, and a gating system signaling to the accumulator that a relevant event is taking place. Arousal is supposed to accelerate the production of pulses of the pacemaker, so that higher arousal corresponds to longer perceived durations, while attention is postulated to drive the gating mechanism^19^. When attention is focused on temporal information, the gate closes and more pulses are stored in the accumulator. As a result, perceived duration is longer. This is particularly true when participants are informed in advance that they have to perform a timing task (prospective timing), since they can allocate more attentional resources in processing temporal information. On the contrary, in retrospective timing, participants incidentally encode temporal information since they are only informed of the timing task once the stimulus has ended. In this situation, time perception mostly relies on memory processes^20–22^. As we have seen before, editing has been shown to modulate attentional allocation, and different kinds of cuts are likely to modulate attentional processes in various manners. Thus, it is likely that cuts signaling a transition between scenes (action discontinuity), and cuts maintaining continuity would have a differential impact on time perception. In particular, cuts signaling an event boundary, like action discontinuity, could transiently increases attention toward visual sensory information and likely divert attention from time processing. In this case, we should predict an underestimation of duration, since temporal information is lost. Nevertheless, there are studies reporting that scenes containing more events are perceived as longer^23,24^. As we have seen, action discontinuities are robust indicators of events boundaries, so we should predict that scenes containing discontinuities should be judged as longer. To our knowledge, no previous study has explored the effect of different types of editing on time perception.

We investigated this question in two studies and employed the same material and editing coding as Magliano and Zacks^13^. We used prospective timing tasks in both studies. Participants were asked to watch short sequences (2500, 3000 and 3500 ms) that either contain no cut, a cut maintaining continuity or a cut introducing discontinuity in time, space and action. Duration was only manipulated to operationalize the timing task. Indeed, always presenting the same duration would most likely have biased participants' responses. Moreover, this manipulation allowed us to check participant accuracy in timing (this was particularly important in the first study conducted online). We did not have any specific hypothesis on the interaction between the two factors (duration and cut type). Participants had to estimate (Study 1) the duration of the scene in an exploratory study conducted online (during the Covid lockdown) or reproduce (Study 2) these durations. They were also asked to judge, after each scene, their level of arousal. Indeed, arousal has been previously proposed to modulate the pulse rate of the pacemaker, and therefore to influence time perception^25^. Finally, as a proxy measure of attentional engagement while watching the excerpts, we introduced a surprise recognition test of snapshots taken from the videos. In Study 1, these two measures (arousal and memory for the snapshots) were only taken as control measures. In particular, given that Study 1 was performed online, the memory measure was added to ensure that participants paid attention, and were able to recognize snapshots extracted from the clips. Thus, no difference between cut conditions was expected on these measures. Moreover, given the contrasting predictions that can be made, particularly on cuts introducing action discontinuities, we did not have any directional hypothesis concerning time estimation in Study 1. In the second pre-registered conceptual replication study conducted in a laboratory setting, we formulated three directional hypotheses based on the results of the Study 1: i) Continuous clips will be associated with longer perceived duration; ii) Continuous clips will be associated with higher levels of self-reported arousal, compared to clips with discontinuities or no editing; 3) Images from continuous clips will be associated with more correct recognitions, followed by images from discontinuous clips, which will be better recognized than images from unedited clips.

## Results

In the following, we report results found with repeated measures ANOVAs from Study 1 and 2 in different sections. In a separate final section, complementary analyses issued from mixed models from both studies are reported.

### Study 1

#### Data analysis

We computed the ‘time estimation error’ by dividing the difference between the time estimation and the real duration by the real duration in milliseconds. Positive value reflects overestimation, while negative values reflect underestimation of the real duration.

We performed repeated-measures ANOVAs on the raw estimation, the time estimation error, and the arousal level with Duration (2500, 3000, and 3500 ms) and Editing type (continuous editing, action discontinuity, and no cut) as factors. A repeated measures ANOVA was also performed for the ratio of correctly recognized images in the recognition task, with Editing type as a factor.

The metric used for effect size is partial eta-squared (η^2^_p_) for ANOVA, and Cohen’s *d* for post-hoc. Post-hoc analyses were conducted using Holm's correction.

Data preprocessing was carried out with *R*^26^ running in R Studio^27^. Inferential statistics were conducted using JASP^28^.

#### Raw estimation

There was a significant main effect of Duration, *F*(2, 178) = 230.62, *p* < 0.001, η^2^_p_ = 0.72. Post-hoc analysis showed that all differences were significant at *p* < 0.001, in particular scenes lasting 2500 ms (marginal mean 2388.31, 95% CI: 2333.81, 2442.81 were judged shorter than scenes lasting 3000 ms (marginal mean 2612.02, 95% CI: 2557.51, 2666.52, Cohen’s d = 1.54) and 3500 ms (marginal mean 2822.89, 95% CI: 2768.39, 2877.39, Cohen’s *d* = 1.81). Scenes lasting 3000 ms were judged shorter than those lasting 3500 ms (Cohen’s *d* =  − 1.18).

There was also a significant main effect of Editing type, *F*(2, 178) = 15.83, *p* < 0.001, η^2^_p_ = 0.15. Post-hoc analysis showed that scenes containing continuous editing (marginal mean 2647.74, 95% CI: 2595.46, 2700.02) were judged longer than both scenes containing action discontinuity (marginal mean 2611.11, 95% CI: 2558.83, 2663.39, *p* = 0.008, Cohen’s *d* = 0.29) and no cut (marginal mean 2564.36, 95% CI: 2512.09, 2616.64, *p* < 0.001, Cohen’s *d* = 0.57). Scenes containing action discontinuity were judged longer than scenes containing no cut (*p* = 0.008, Cohen’s *d* =  − 0.31).

The interaction between the two factors was not significant, *F*(4, 356) = 1.06, *p* = 0.37, η^2^_p_ = 0.01.

#### Time estimation error

There was a significant main effect of Duration, *F*(2, 178) = 296.29, *p* < 0.001, η^2^_p_ = 0.77. Post-hoc analysis showed that all differences were significant at *p* < 0.001. Scenes lasting 2500 ms (marginal mean − 0.04, 95% CI: − 0.06, − 0.03) were associated with a smaller estimation error than scenes lasting 3000 ms (marginal mean − 0.13, 95% CI: − 0.15, − 0.11, Cohen’s *d* = 1.71) and 3500 ms (marginal mean − 0.19, 95% CI: − 0.21, − 0.18, Cohen’s *d* = 2.10). Scenes lasting 3000 ms were associated with a smaller estimation error than those lasting 3500 ms (Cohen’s *d* = 1.24).

There was also a significant main effect of Editing type, *F*(2, 178) = 16.39, *p* < 0.001, η^2^_p_ = 0.16. Post-hoc analysis showed that scenes containing continuous editing (marginal mean − 0.11, 95% CI: − 0.13, − 0.09) were associated with a smaller estimation error than scenes containing action discontinuity (marginal mean − 0.12, 95% CI: − 0.14, − 0.10, *p* = 0.005, Cohen’s *d* = 0.33) and no cut (marginal mean − 0.14, 95% CI: − 0.15, − 0.12, *p* < 0.001, Cohen’s *d* = 0.58). Scenes containing action discontinuity were associated with a smaller estimation error than those containing no cut (*p* = 0.006, Cohen’s *d* =  − 0.29).

The interaction between the two factors was not significant, *F*(4, 356) = 1.39, *p* = 0.24, η^2^_p_ = 0.01.

#### Arousal

There was a significant main effect of Duration, *F*(2, 178) = 5.39, *p* = 0.005, η^2^_p_ = 0.06. Post-hoc analysis showed that scenes lasting 2500 ms (marginal mean 3.19, 95% CI: 2.86, 3.51) were associated with lower reported arousal than scenes lasting 3000 ms (marginal mean 3.31, 95% CI: 2.99, 3.64, *p* = 0.010, Cohen’s *d* =  − 0.32) and 3500 ms (marginal mean 3.33, 95% CI: 3, − 3.66, *p* = 0.02, Cohen’s *d* =  − 0.28). The difference between scenes lasting 3000 and 3500 ms was not significant (*p* = 0.68, Cohen’s *d* =  − 0.04).

There was also a significant main effect of Editing type, *F*(2, 178) = 18.16, *p* < 0.001, η^2^_p_ = 0.17. Post-hoc analysis showed that scenes containing continuous editing (marginal mean 3.42, 95% CI: 3.10, 3.75) were associated with higher reported arousal than scenes containing action discontinuity (marginal mean 3.20, 95% CI: 2.88, 3.52, *p* < 0.001, Cohen’s *d* = 0.48) and no cut (marginal mean 3.21, 95% CI: 2.88, 3.53, *p* < 0.001, Cohen’s *d* = 0.58). Scenes containing action discontinuity and no cut did not differ (*p* = 0.87, Cohen’s *d* = 0.02).

The interaction between the two factors was not significant, *F*(4, 356) = 1.21, *p* = 0.3, η^2^_p_ = 0.01.

#### Recognition

There was a significant main effect of Editing type, *F*(2, 178) = 22.92, *p* < 0.001, η^2^_p_ = 0.2. Post-hoc analysis showed that images extracted from scenes containing no cut (mean = 0.74, SD = 0.16) were better recognized than both images extracted from scenes containing continuous editing (mean = 0.68, *SD* = 0.18, *p* < 0.001, Cohen’s *d* =  − 0.4) and action discontinuity (mean = 0.62, *SD* = 0.17, *p* < 0.001, Cohen’s *d* = 0.7). Images extracted from scenes containing continuous editing were better recognized than those presenting action discontinuity (*p* = 0.003, Cohen’s *d* = 0.32).

### Study 2

#### Data analysis

We computed the ‘time reproduction error’ by dividing the difference between the time reproduction and the real duration by the real duration in milliseconds. Positive value reflects overestimation, while negative values reflect underestimation of the real duration. The analysis carried out for this study was identical to Study 1.

#### Raw reproduction

There was a significant main effect of Duration, *F*(2, 118) = 126.07, *p* < 0.001, η^2^_p_ = 0.68. Post-hoc analysis showed that all differences were significant at *p* < 0.001. In particular, scenes lasting 2500 ms (marginal mean 2587.78, 95% CI: 2366.80, 2808.75) were reproduced shorter than scenes lasting 3000 ms (marginal mean 2910.70, 95% CI: 2689.72, 3131.67, Cohen’s *d* = − 1.07) and 3500 ms (marginal mean 3294.86, 95% CI: 3073.88, 3515.83, Cohen’s *d* = − 1.98). Scenes lasting 3000 ms were reproduced shorter than those lasting 3500 ms (Cohen’s *d* = − 1.03).

There was also a significant main effect of Editing type, *F*(2, 118) = 15.04, *p* < 0.001, η^2^_p_ = 0.2. Post-hoc analysis showed that scenes containing continuous editing (marginal mean 3030.49, 95% CI: 2811.05, 3249.94) were reproduced longer than both scenes containing action discontinuity (marginal mean 2939.93, 95% CI: 2720.48, 3159.37, *p* = 0.011, Cohen’s *d* = 0.31) and no cut (marginal mean 2822.91, 95% CI: 2603.46, 3042.35, *p* < 0.001, Cohen’s *d* = 0.76). Scenes containing action discontinuity were judged longer than scenes containing no cut (*p* = 0.011 Cohen’s *d* = − 0.37).

The interaction between the two factors was not significant, *F*(4, 236) = 0.38, *p* = 0.82, η^2^_p_ = 0.006.

#### Time reproduction error

There was a significant main effect of Duration, *F*(2, 118) = 21.59, *p* < 0.001, η^2^_p_ = 0.27. Post-hoc analysis showed that scenes lasting 2500 ms (marginal mean − 0.03, 95% CI: − 0.04, 0.11) were associated with a positive reproduction error that differed significantly from scenes lasting 3000 ms (marginal mean − 0.03, 95% CI: − 0.10, 0.04, *p* < 0.001, Cohen’s *d* = 0.56) and 3500 ms (marginal mean − 0.06, 95% CI: − 0.13, 0.02, *p* < 0.001, Cohen’s d = 0.81). Scenes lasting 3000 ms were associated with a smaller estimation error than those lasting 3500 ms (*p* = 0.44, Cohen’s d = 0.26).

There was also a significant main effect of Editing type, *F*(2, 118) = 16.52, *p* < 0.001, η^2^_p_ = 0.22. Post-hoc analysis showed that scenes containing continuous editing (marginal mean 0.02, 95% CI: − 0.06, 0.09) were associated with a more positive reproduction error than scenes containing action discontinuity (marginal mean − 0.02, 95% CI: − 0.09, 0.06, p = 0.011, Cohen’s d = 0.34) and no cut (marginal mean − 0.05, 95% CI: − 0.13, 0.02, *p* < 0.001, Cohen’s *d* = 0.79). Scenes containing action discontinuity were associated with a smaller negative reproduction error than those containing no cut (*p* = 0.010, Cohen’s *d* = − 0.38).

The interaction between the two factors was not significant, *F*(4, 236) = 0.72, *p* = 0.58, η^2^_p_ = 0.01.

#### Arousal

There was a significant main effect of Editing type, *F*(2, 118) = 12.28, *p* < 0.001, η^2^_p_ = 0.17. Post-hoc analysis showed that scenes containing continuous editing (marginal mean 3.08, 95% CI: 2.70, 3.455) were associated with higher reported arousal than scenes containing action discontinuity (marginal mean 2.93, 95% CI: 2.55, 3.30, *p* = 0.005, Cohen’s *d* = 0.41) and no cut (marginal mean 2.88, 95% CI: 2.51, 3.25, *p* < 0.001, Cohen’s *d* = 0.61). Scenes containing action discontinuity and no cut did not differ (*p* = 0.17, Cohen’s *d* = − 0.18).

The main effect of Duration, *F*(2, 118) = 0.84, *p* = 0.43, η^2^_p_ = 0.01, and the interaction between the two factors were not significant, *F*(4, 236) = 0.74, *p* = 0.57, η^2^_p_ = 0.01.

#### Recognition

There was a significant main effect of Editing type, *F*(2, 118) = 3.17, *p* = 0.046, η^2^_p_ = 0.05. Post-hoc analysis do not reveal any significant differences. Images extracted from scenes containing no cut (mean = 0.74, SD = 0.15) were not better recognized than both images extracted from scenes containing continuous editing (mean = 0.71, *SD* = 0.13, *p* = 0.29, Cohen’s *d* =  − 0.19) and action discontinuity (mean = 0.69, *SD* = 0.17, *p* = 0.06, Cohen’s *d* = 0.31). Images extracted from scenes containing continuous editing were not better recognized than those presenting action discontinuity (*p* = 0.29, Cohen’s *d* = 0.14).

### Complementary analyses: mixed models

**Figure 1:**
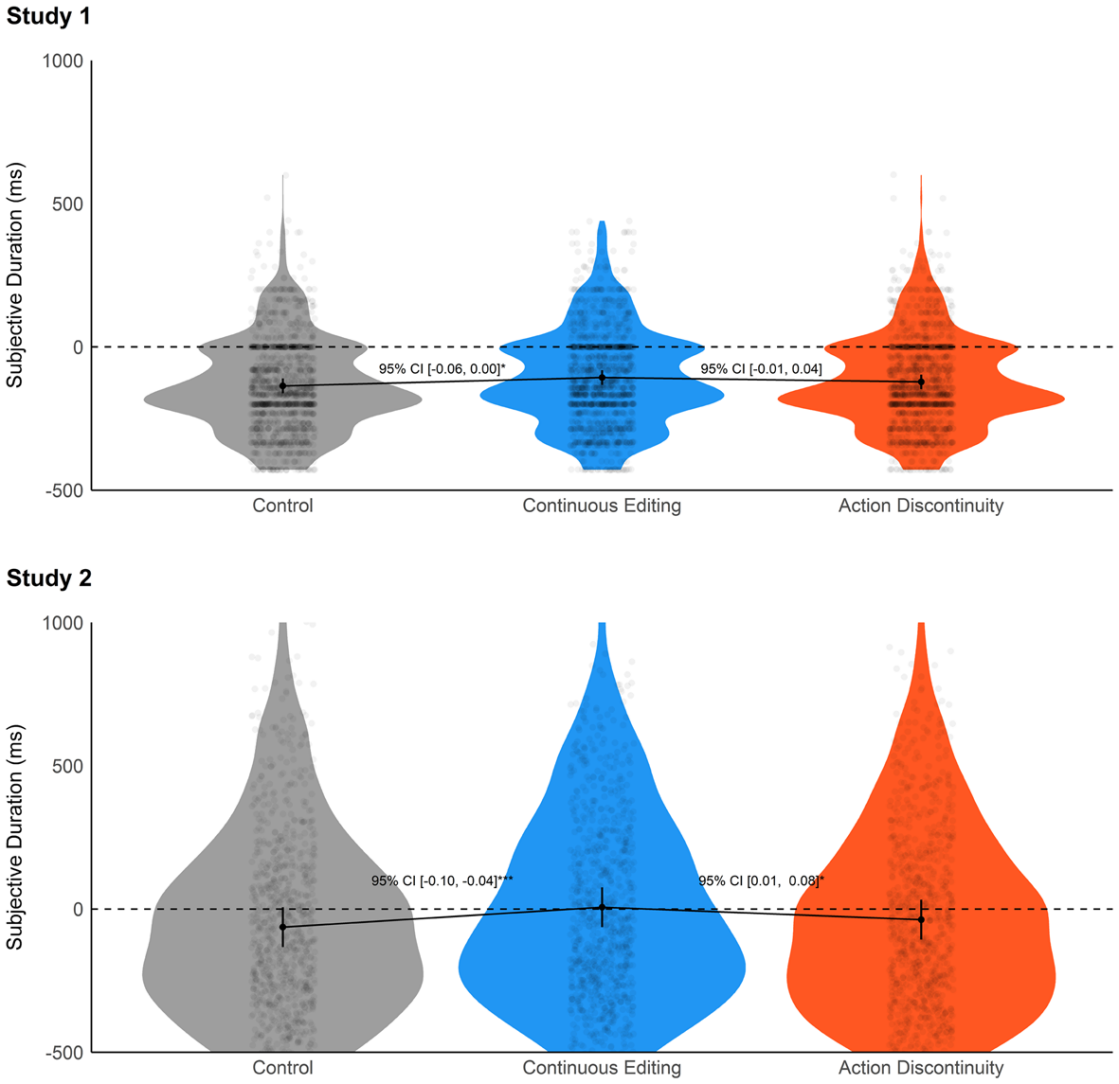
Time estimation error for the three editing types. The picture represents the marginal means (estimated by mixed models) with data distributions and data points. Error bars represents 95% CI. The confidence intervals of marginal differences between conditions are reported between the conditions of interest. *p < 0.05, ***p < 0.001.

Analysis was carried out using R 4.2^29^, using the glmmTMB (Brooks and et al., 2017) and easystats packages^30–32^. We report here only the main results on time estimation error (judgement and reproduction, respectively for Study 1 and 2; see Fig. 1), arousal, and recognition. Complete results are available here: https://osf.io/eb6r9/files/osfstorage.

#### Time estimation error

Time estimation error was modelled using linear mixed regressions with participants and items set as random factors, and Editing type (continuous editing, action discontinuity, and no cut) as fixed factor. In Study 1, marginal contrast analysis revealed that continuous editing was related to longer reported durations as compared to the control condition (marginal difference =  − 0.03, 95% CI: − 0.06, 0.00, *p* = 0.049). The difference between action discontinuity and continuous editing was not significant (marginal difference = 0.02, 95% CI: − 0.01, 0.04, *p* = 0.298).

In Study 2, marginal contrast analysis revealed that continuous editing was related to longer reported durations compared to the control condition (marginal difference =  − 0.07, 95% CI: − 0.10, − 0.04, *p* < 0.001), and that the difference between action discontinuity and continuous editing was also significant (marginal difference = 0.04, 95% CI: 0.01, 0.08, *p* = 0.01).

#### Arousal

In Study 1, adding the subjective arousal as predictor confirmed that continuous editing was related to longer reported durations as compared to the control condition (marginal difference = 0.04, 95% CI: 0.05, 0.07, *p* = 0.023). Moreover, this model revealed a positive and significant effect on the time estimation error in the control condition (beta = 0.01, 95% CI: 0.00, 0.02, *p* < 0.001), The interaction with continuous editing condition (beta =  − 0.004, 95%: CI: − 0.009, 0.002, *p* = 0.161), and action discontinuity condition (beta =  − 0.004, 95% CI: − 0.009, 0.002, *p* = 0.007) was not significant, showing a similar effect of arousal in all conditions.

In Study 2, adding the subjective arousal as predictor confirmed that continuous editing was related to longer reproduced durations as compared to the control condition (marginal difference = 0.07, 95% CI: 0.02, 0.12, *p* = 0.004). Moreover, the model showed that there was no effect of arousal in the control condition (beta =  − 0.005, 95% CI: − 0.02, 0.00, *p* = 0.351), nor an interaction with the continuous editing (beta =  − 0.001, 95% CI: − 0.01, 0.01, *p* = 0.884) or the action discontinuity condition (beta = 0.005, 95% CI: − 0.003, 0.02, *p* = 0.451), showing no link between arousal and time reproduction.

#### Recognition

Logistic mixed models suggested that items presented in the action discontinuity condition were less recognized compared to the control condition in Study 1 (marginal difference =  − 0.60, 95% CI: − 0.01, − 1.20, *p* = 0.046). No other effects were significant. No significant differences emerged in Study 2.

## Discussion

The main goal of the two present studies was to test whether different types of cuts, in particular cuts representing action continuity within a scene and cuts leading to discontinuity, differentially modulate spectators’ time perception. The results of the two studies were coherent, with results of Study 1 replicated by Study 2 (pre-registered). Overall, the results showed that video excerpts containing both types of editing were perceived as longer than those containing no cuts. Moreover, scenes containing continuous cuts were also perceived as longer than those presenting discontinuity. This finding was also replicated by more stringent complementary analysis using mixed models, thus confirming our first preregistered hypothesis. We also reported that videos with continuous editing were judged as more arousing than the other two kinds of scenes, and that snapshots extracted from unedited scenes were in general better recognized than those taken after cuts in both continuous and discontinuous editing, even if these last results were less robust across the two studies (no differences were reported in Study 2).

The finding that scenes containing editing were perceived as longer that those without cuts are partially in line with the results of Balzarotti et al.^12^ showing that the density of editing (number of cuts) was associated with an overestimation of video duration compared with unedited video. Nevertheless, they only reported a significant difference between fast-paced editing (10–12 cuts) and unedited scenes, but the difference between the latter and slow-paced (5 cuts) editing was not significant. On the contrary, here we reported significant differences between scenes containing only one cut and those with no cut. Of note is that in the study by Balzarotti et al.^12^, the difference between slow-paced and unedited scenes went in the same direction and approached significance (*p* = 0.056), suggesting that the null results could be due to low statistical power. In any case, the comparison between the two studies is not straightforward due to methodological differences. For example, in Balzarotti et al.^12^ durations were in the order of tenth of seconds (11–13.5 s), while we used durations between 2500–3500 ms. Our results are coherent with recent proposals that tracking changes in low-level perceptual processing provides a basis for human time perception^33,34^, in particular with results showing that naturalistic videos with greater perceptual change were estimated as longer in duration^33^. Indeed, even if we did not directly measure perceptual change, a cut introduces an abrupt change in the visual information flow by definition.

If perceptual changes would solely predict time perception, we should expect perceived longer duration for discontinuous scenes. According to Magliano and Zacks^13^, a stimulus-driven increase of processing and an attention-driven modulation mechanism operate at points of discontinuity corresponding to cuts. Continuous editing would be expected to produce larger stimulus-driven increases in processing that may contribute to bridging the perceptual discontinuities to maintain continuity. Crucially, this process would be superfluous for discontinuity cuts as they signal a major scene change. In this case, top-down attentional down-regulation would suppress the additional processing driven by the presentation of new visual information. This kind of pattern of activity, hyper-activation for continuous and de-activation for discontinuous cut, was reported in a set of brain regions encompassing the non-primary visual cortex, inferotemporal cortex, and parietal cortex^13^. We speculatively argue that this additional neural activity could explain the results we reported here. Further neuroimaging studies are needed to confirm this hypothesis.

In the present study continuous editing was associated with higher self-reported arousal, compared to the other two conditions, confirming our second preregistered hypothesis. This finding resembles the pattern of results for the perceived duration, and is coherent with the literature showing that higher arousal is associated with longer perceived duration^35^. Nevertheless, we believe that this result should be taken with caution. Indeed, even if scenes with continuous editing were judged to be more arousing, their overall level of arousal is quite low in both studies (on average 3.42 and 3.08 in Study 1 and 2 respectively on a scale ranging from 1 to 9). Even if complementary analysis (mixed models) showed that higher arousal predicted longer perceived durations, this was true in all conditions. Additionally, the difference in perceived time between continuous cut and the control condition remained significant in this model, suggesting that the effect of cut types was not explained by arousal. Moreover, the link between arousal and perceived duration was only significant in Study 1. Taken together, these results are coherent with recent findings questioning the role of arousal in time perception, above all for complex visual stimuli. Indeed, Suárez-Pinilla, et al.^33^ did not find any relationship between autonomic response (heart rate) and duration estimation when participants observed naturalistic video stimuli.

Finally, qualitatively we reported a similar pattern of results in both studies concerning memory performance that we meant as a proxy measure of attention while watching videos. Snapshots extracted from unedited scenes were better recognized than those extracted after cuts, even if this effect failed to reach significance in the second study. Mixed models confirmed that the probability of recognizing a snapshot was significantly lower in the discontinuity editing compared to the control condition only in the first study. No difference emerged in the second study. For the first study, the results are likely due to the choice of the material and did not reflect the general attentional engagement during viewing. Indeed, we chose to take snapshots for the recognition task just after the cut (1 frame in the Study 1). Shimamura, et al.^10^ showed that detection accuracy of targets (asterisk) presented during a video clip decreased when the target was presented just after an edit, compared with a target presented before the edit or in the middle of a shot. This result suggests that cuts could temporarily capture attention and disrupt processing and subsequent encoding of information presented in close succession. This explanation is corroborated by the absence of differences in Study 2, in which we choose, for the recognition task, snapshot that were more shifted in time from the cut (5–10 frames). Future studies should combine more direct behavioral and neurophysiological measures of attention to investigate the interplay between editing, attentional regulation and time perception.

To resume, editing modulates time perception, leading to longer perceived duration compared to unedited videos. In particular, cuts allowing narrative continuity, despite perceptual discontinuity, showed the strongest effect on time perception. Editing is a series of techniques that impose a structure on a visual flow of information to allow narrative coherence and facilitate comprehension. This seems to be exactly what our perceptual system does in order to deal with the continuous stream of information. Indeed, following the Event Segmentation Theory, the perceptual system automatically parses in time the flow of information in discrete events^14,15^. This facilitates the perceptual and cognitive organization of information, allowing the system to efficiently and transiently allocate attentional resources to pertinent information, to structure it and make sense of it, resulting in a mnemonic advantage for information that has been properly segmented. Despite the numerous studies investigating the impact of event segmentation on attentional and memory processes, a surprising few investigate the impact of segmentation on time perception^23,24,36^. In an initial study, Bangert, et al.^23^ asked participants to reproduce a previously encoded duration while watching naturalistic videos containing many (eventful), few (uneventful) event boundaries or a blank screen. The authors showed that reproduction was shorter for eventful videos compared to the other two conditions, and for uneventful videos compared to the blank condition. Similar results were found by Fenerci, et al.^24^ reporting that videos containing an single event boundary (spatial shift) were reproduced as shorter than target durations, compared to videos not containing boundaries (steady-cam shot), and that the duration of the same videos was retrospectively judged as longer (see also^37^). Taken together, these results suggest that the number of events (spatial shifts or cuts) makes participants perceive that more time has passed. Even if these findings are generally coherent with our main results that scenes containing cuts are perceived as longer than scenes without editing, one would expect that clips containing discontinuous editing would be perceived as longer than those containing continuous cuts. Indeed, as shown by Magliano and Zacks^13^, discontinuous editing, contrary to continuous cuts, is a robust predictor of event perception. Nevertheless, it has to be noted that in Bangert, et al.^23^ eventfulness was established on participants’ behavioral segmentation of scenes of everyday activity not containing cuts. In the same vein, in Fenerci, et al.^24^ events were defined as a shift in spatial context (e.g., characters moving through doorways). This last situation resembles continuous editing since temporal and action continuity are maintained. Neither of the two studies contained a condition similar to discontinuous editing. Moreover, making a direct comparison between these studies and our work is not straightforward due to important methodological differences concerning the material, the range of the estimated duration, and the timing task. On the contrary, in a subsequent study, Bangert, et al.^36^ reported that the duration of test intervals presented across a single event boundary while watching videos was more frequently judged as shorter than a target duration, compared to test intervals presented within an event (the target and the test intervals had always the same duration of 5 s). Discrepancies in the results are likely due to methodological differences including material used, durations, as well as instructions provided to the participants (e.g. reproduction, estimation). Overall, our contribution adds information on how different types of events (visual continuous or discontinuous cut) modulate time perception and gives important insight on how studying movie editing could be pertinent for our understanding of time perception in real life under the Event Segmentation framework. Moreover, the present findings are of interest to better apprehend how film editing, and in general cinema formalism (camera movements etc.), influences time perception by providing valuable clues as to how time perception is impacted by natural phenomena producing similar visual consequences (e.g. eye movements, blinks)^38^. Finally, this study contributes to the emerging interdisciplinary field of psycho-cinematics which could ultimately develop the dialog between arts and science^1^, by questioning how artists’ intuitions could be considered in experimental psychology.

## Methods

### Study 1

#### Participants

One hundred and thirty-three participants were recruited during the first Covid-19 lockdown (autumn-spring 2020) among the undergraduate students at the Institute of Psychology at Université Paris Cité using an announcement on the university portal. Participants received class credit for their participation. Participants were not familiar with the film. Forty-three participants were excluded according to the following criteria: reporting a history of neurological or psychiatric disorders (9 participants); having used alcohol or drugs before the experiments (6 participants); having been interrupted during the protocol (16 participants); having already seen the movie (1 participants); having completed the protocol with a duration longer than 1.96 SD (6 participants), having an A’ on the recognition task equal or inferior to 0.5 (2 participants); being over 40 years old (3 participants). A’ is a non-parametric index of discriminability of the signal detection theory (SDT). This index is preferred to the parametric index d’ since it is not dependent on assumptions about the distribution of signal and noise, and is not sensitive to extreme values of Hit and False alarms rates^39^. The final sample was composed of 90 participants (77.78% females, mean age 20.31 ± 1.85 years).

All participants were informed of the academic nature of the study and accepted that their responses would be processed anonymously. Then, all participants gave written informed consent before carrying out the study. The protocol was carried out in accordance with local ethical standards.

#### Material

**Figure 2:**
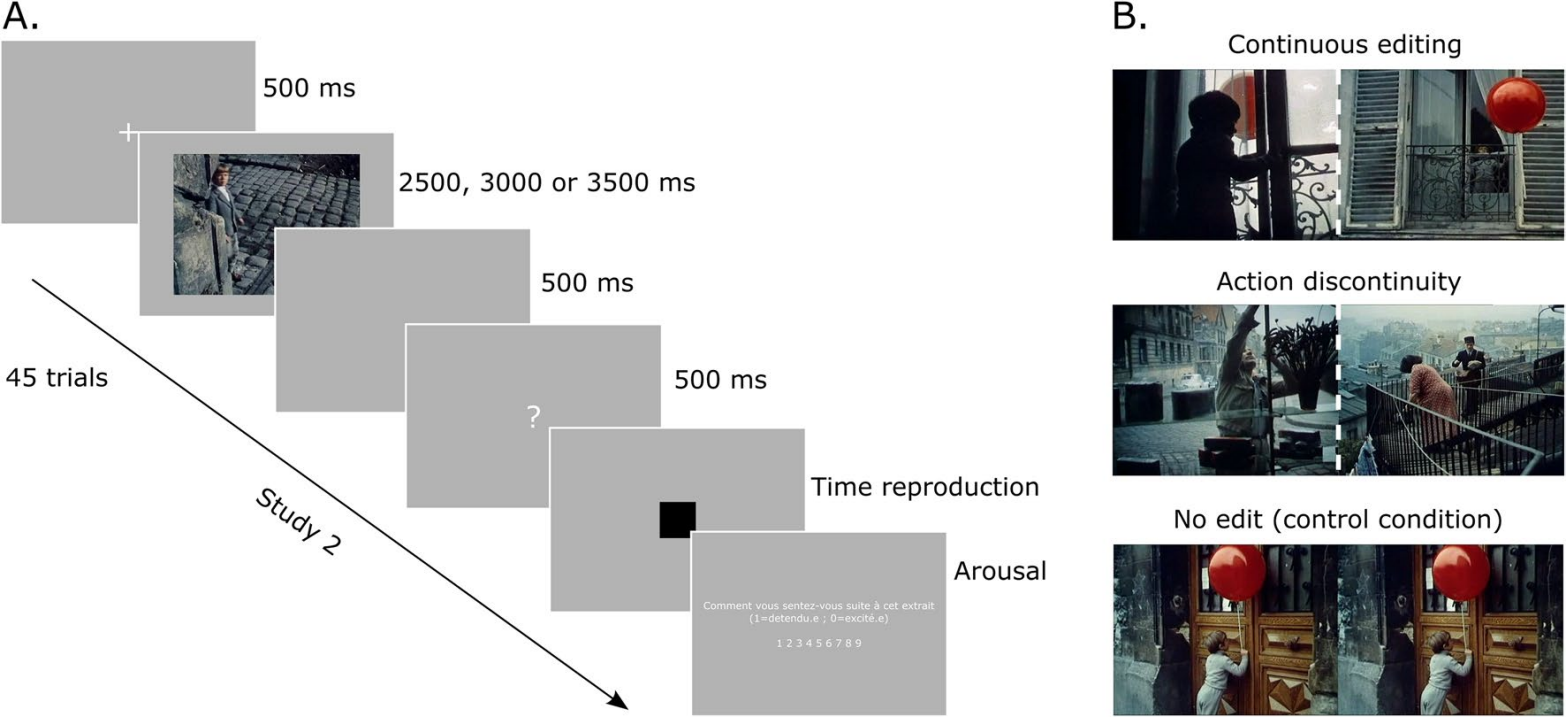
(**A**) Example of time course for Study 2. Stimuli were presented after a fixation cross (500 ms) for one of the three durations (2500, 3000 or 3500 ms). Then, an empty screen (500 ms) followed by a question mark (500 ms) were shown before the appearance of a black square at the center of the screen. Participants had to press the spacebar when they estimated that the duration of presentation of the square was equivalent to the duration of the preceding video clip. After the time reproduction, an arousal scale was presented. (**B**) Examples of edits for each condition. In the continuous condition, two edited sequences show the same action although a change of perspective (a child looking out of the window), while in the action discontinuity condition the edited sequences present two separate actions. In the control condition the were no edits.

We selected excerpts from the movie *Le Ballon Rouge* (Lamorisse, 1956), and extracted 45 unique sequences, 15 in the continuous editing condition, 15 in the action discontinuity editing condition and 15 with no cut, according to the editing coding of Magliano and Zacks^13^. Each sequence was formatted in 3 durations: 2500 ms, 3000 ms, and 3500 ms. For edited clips, the different durations were obtained by manipulating the time window around the time of the cut (e.g. for 2500 ms, we took 1250 ms before and after the cut). For unedited clips, we used the same procedure, but the time window was centered around a reference frame that was hold constant for the three durations (see Fig. 2).

For the recognition task we extracted the image corresponding to the frame following the cut for the sequences containing editing, and the central frame for the sequences without editing. We also extracted 30 images from other sequences of the movie to be used as lures.

The Metacognitive Questionnaire on Time was also employed as an interference task between the time estimation and the recognition task^40^. This task created a retention interval before the recognition task. This questionnaire is composed of 12 items rated on a 5-point Likert scale. In the present study, the scores from this questionnaire were not used further in the analyses.

#### Time estimation task

The task began with a brief synopsis of the movie, followed by instructions. Participants were instructed to try to estimate the duration of each sequence as accurately as possible without using external devices. They were also notified that, after each time estimation, they would be asked to judge their emotional reaction to each sequence.

Each trail started with the presentation of a sequence that was immediately followed by the appearance of a scale ranging from 2000 ms and 4000 ms with intervals of 100 ms. The participants had to use their mouse to select the estimated duration. We used an interval longer than the actual presented durations, since it has been shown that participants tend to avoid using the extremes of a scale, and that this modification can counteract this bias, leading to more precise estimation^41^. The participants were asked not to use any temporal support to perform the task, nor to count. Then, participant had to estimate their arousal on a 9-point Likert scale (1-relaxed, 9-excited). Afterwards a new trial began.

The 45 sequences were presented in a randomized order. The association between the editing condition and the duration was counterbalanced across participants so that each sequence was presented in all three durations in the whole sample, but each participant saw a specific sequence once.

#### Recognition task

Participants were instructed that they would see a list of pictures belonging to the sequences presented in the previous task, and other pictures extracted from the same movie, but that were not presented before. They had to indicate if they had already seen the scene represented in the picture or not.

Each trial began with the presentation of one picture, and participants had to click on a ‘yes’ or ‘no’ button with their mouse. The presentation was self-paced, so that after participants’ response, a new trial began. The 45 target pictures and the 30 lures were presented in a randomized order.

#### Procedure

Due to the Covid-19 pandemic the study was conducted completely on-line. The protocol was programmed on Psytoolkit^42,43^. The link to access the original (French) version of the Time Estimation Task is the following: https://osf.io/eb6r9/files/osfstorage.

Participants were requested to pass the experience in a quiet place to avoid distraction. They were informed they would watch different video sequences and they would be asked to estimate their duration, and that they would have to answer some questionnaires.

After having read the protocol description and after accepting to take part in the study, they responded to socio-demographic questions (age, sex) and completed the PHQ-4 questionnaire^44^. Participants completed the Time estimation task, followed by the Metacognitive Questionnaire on Time^40^, and the Recognition task. The protocol ended with questions assessing the participants’ habits toward films (e.g., the frequency of movie watching, preferred movie style), if they knew the film from which the sequences were extracted, and their editing experience.

The total duration of the protocol was 16.73 ± 3.8 min.

### Study 2

#### Participants

We used the software program G*Power to conduct a power analysis. Our goal was to obtain 0.8 power to detect an effect size of 0.43, based on the effect size obtained in our first exploratory study concerning the effect of Editing on the Time estimation error, at the standard 0.05 alpha error probability. Our subsequent target sample size was 56 participants. We recruited 70 participants among the undergraduate students at the Institute of Psychology at Université Paris Cité using an announcement on the university portal. Participants received class credit for their participation. Participants were excluded according to the following criteria: reporting a history of neurological or psychiatric disorders (8 participants); incomplete data due to technical reasons (2 participants). None of the participants were excluded for having an A’ inferior to 0.5. The final sample was composed of 60 participants (95% females, mean age 19.78 ± 3.12 years), and no participant watched the film before the task.

All participants were informed of the academic nature of the study and accepted that their responses would be processed anonymously. After the nature of the procedure had been fully explained, all participants gave written informed consent before carrying out the study. The protocol was approved by the local ethical committee (Université Paris Cité, IRB number: 0012022–5).

#### Material

The material was identical to Study 1, except the images employed for the recognition task. Indeed, in this study the images from the edited sequences (continuous editing and action discontinuity) were captured 5 frames (167 ms) after the cut. For two sequences only, the images were taken 10 frames (333 ms) after the cut, in order to have a salient element in the image.

#### Time reproduction task

The task began with a brief synopsis of the movie, followed by the instructions. Participants were instructed that they would have to reproduce the duration of a movie sequences, as accurately as possible without using external devices, by ending the presentation of a visual stimulus when they estimate it is comparable to that of the previous sequence. They were also told that, after the time estimation, they would be asked to judge their emotional reaction to each sequence.

Following the time reproduction procedure in Damsma, et al.^41^, each trail started with a fixation cross lasting 500 ms that was followed by the presentation of a sequence. At the end of the sequence an empty screen was presented for 500 ms, followed by a question mark at the center of the screen for 500 ms that served to indicate the reproduction task. This was followed by a black square (216 × 216 pixel) presented at the center of the screen. Participants had to click on the spacebar to stop the presentation of the square when they estimated that the duration of presentation was equivalent to that of the preceding sequence. Then, participant had to estimate on a 9-point Likert scale their arousal (1-relaxed, 9-excited). A new trial began afterwards.

The 45 sequences were presented in a randomized order. The association between the editing condition and the duration was counterbalanced across participants so that each sequence was presented in all three durations in the whole sample, but each participant saw a specific sequence once.

The main task was preceded by a familiarization phase of 10 trials that followed the procedure of the experimental task, excepting the stimuli whose duration had to be reproduced and the duration employed. In the familiarization phase, the stimuli consisted in 30 randomly moving dots on a rectangular background that were presented during 2000 ms, 2500 ms, 3000 ms, 3500 ms, and 4000 ms (each duration was presented twice).

#### Recognition task

Study 2 followed the same procedure as the Recognition task in Study 1, except for the stimuli employed (see Fig. 2).

#### Procedure

The experimental sessions took place in an experimental room at the Institute of Psychology of Université Paris Cité. The protocol was programmed with PsychoPy 3^45^, and was delivered on a computer screen Iiyama G-master GE2488HS (568 × 409 × 217.5 mm, 1920 × 1080 pixel resolution, 75 Hz). Participants were seated at about 60 cm from the screen.

After the protocol description had been explained, all participants signed the informed consent, and answered socio-demographic question (age, sex). Participants completed the Time reproduction task, followed by the Metacognitive Questionnaire on Time^40^, and the Recognition task. The protocol ended with questions assessing the participants’ habits toward movies (e.g., the frequency of movie watching, preferred movie style), if they knew the movie from which the sequences were extracted, and their editing experience. As for Study 1, only participants that did not know the film were included in the analyses.

The study was publicly preregistered on the Open Science Framework repository (https://osf.io/vhxa9).

### Ethics approval

This research was conducted in accordance with the local ethical standards and the Declaration of Helsinki. The conceptual replication study (Study 2) has been publicly preregistered on the Open Science Framework repository (https://osf.io/vhxa9) and received local ethical approval by Université Paris Cité (IRB number: 0012022–5).

## Data Availability

The datasets generated during and/or analyzed during the current study are available from the corresponding author on reasonable request.
